# Predictive Modelling in Clinical Bioinformatics: Key Concepts for Startups

**DOI:** 10.3390/biotech11030035

**Published:** 2022-08-17

**Authors:** Ricardo J. Pais

**Affiliations:** 1 Bioenhancer Systems, Office 63 182-184 High Street North, East Ham, London E6 2JA, UK; rjpais@bioenhancersystems.com; 2 Centro de investigação Interdisciplinar Egas Moniz (CiiEM), Instituto Universitário Egas Moniz, 2829-511 Caparica, Portugal

**Keywords:** predictive modelling, clinical bioinformatics, mathematical models, diagnostics, prognostics, clinical applications

## Abstract

Clinical bioinformatics is a newly emerging field that applies bioinformatics techniques for facilitating the identification of diseases, discovery of biomarkers, and therapy decision. Mathematical modelling is part of bioinformatics analysis pipelines and a fundamental step to extract clinical insights from genomes, transcriptomes and proteomes of patients. Often, the chosen modelling techniques relies on either statistical, machine learning or deterministic approaches. Research that combines bioinformatics with modelling techniques have been generating innovative biomedical technology, algorithms and models with biotech applications, attracting private investment to develop new business; however, startups that emerge from these technologies have been facing difficulties to implement clinical bioinformatics pipelines, protect their technology and generate profit. In this commentary, we discuss the main concepts that startups should know for enabling a successful application of predictive modelling in clinical bioinformatics. Here we will focus on key modelling concepts, provide some successful examples and briefly discuss the modelling framework choice. We also highlight some aspects to be taken into account for a successful implementation of cost-effective bioinformatics from a business perspective.

## 1. Clinical Bioinformatics Role and its Dependency on Predictive Modelling

Clinicians consider access of patients’ genetic information from genomes, transcriptomes, proteomes and metabolomes as advantageous for improving diagnostics and prognostics of diseases [1,2,3,4,5]. Accessing clinically relevant information from these ‘omics’ data is considered by many as precision medicine, which has the potential to enable more personalized and effective medicine [1,4,5]. Current advances in Next Generation Sequencing (NGS) and Mass Spectrometry (MS) technologies made possible the characterization of genomes and quantification of proteomes from patients’ biological samples with reasonable accuracy and scalability, compatible with its application in the clinical point-of-care [6,7,8,9,10,11]; however, data from these technologies is too complex to be humanly handled and interpreted by clinicians. Bioinformatics is fundamental for providing humanly readable and clinically relevant genomics and proteomics interpretations from NGS and MS techniques [1,3,8,9]. For these reasons, bioinformatics is considered as a fundamental bridge between clinicians and ‘omics’ technology making this field quite attractive to the medical community. Another attractive feature of bioinformatics is also due to its potential to facilitate the automation of data analysis and opens the possibility for “big data” processing [12,13,14,15]; this will be advantageous when the new digital Era fully reaches the medical industry and clinical laboratories [7,16]. Although bioinformatics has its origins in evolutionary biology and intimately linked to genomics, a new clinically focused branch is growing fast and diversifying from the traditional bioinformatics [3,17]; this is called by many clinical bioinformatics and its objectives are focused on obtaining diagnostics, prognostics, or therapy assessment out of the data from an individual patient. The clinical bioinformatics branch is expected to play a central role in facilitating the identification of genetic diseases, the discovery of novel biomarkers, characterization of pathogens and enable a more informed decision for the therapeutical strategy to follow [3,18,19].

Predictive modelling on the other hand is a speciality commonly used in data science, computational biology and systems biology for more than six decades [5,20]. Recently, researchers have been proposing the combination of predictive modelling approaches with bioinformatics for improving current practices in disease identification, therapeutics and prognostics [13,18,21,22,23]; these have been shown advantageous to unlock the full potential of many high-throughput technologies as solutions for large population screening of multiple disorders and precision medicine; this is evident for high-throughput technologies such as mass spectrometry and next-generation sequencing which contains a huge and complex amount of information that require both high computer processing power and advanced mathematical modelling approaches for translating the complexity of the data into clinically relevant predictions [5,13,21]. Therefore, predictive modelling is set to play a key role in clinical bioinformatics, which should become part of the standard clinical bioinformatic pipelines as a downstream analysis step following a traditional bioinformatics pipeline; however, this step needs to be integrated with typical bioinformatics pipelines from genomics, proteomics and transcriptomics. In each of these cases, the predictive modelling step takes as input the bioinformatically curated “omics” data, integrates it with other sources of patient data (metadata) and generates an output that should be relevant phenotype information readable by a clinician; this integration is not an easy task and would depend on high-throughput data, the available metadata, the target disease and more important the choice of the modelling framework.

## 2. Key Concepts of Predictive Modelling in the Clinical Context

Despite the efforts for conveying the correct role of predictive modelling in the clinical context, there is still some misunderstanding in the medical and biotech community regarding key concepts underlying its application [18,24,25,26]; this often results in either undervaluing the modelling step or extrapolating it to a science fiction story. To fully understand the clinical applications of predictive modelling and their caveats, we should start by simply defining what is a predictive model in the first place. A predictive model is any mathematical abstraction of a system which generates a prediction of an unknown system component/property based on known system components [24,27,28]. In our case, the model is a conceptual description of a biomedical system of interest, where the components of the system are:

- Biological relevant and measurable or observational entities (dependent variables), which are the inputs of the model.
- Relational factors between variables with or without biological meaning (parameters), which can be estimated empirically or based on data fitting methodologies.
- Unknown clinical entities or properties of interest for prediction (dependent variables), which are the outputs of the model.

Models can be formally translated through mathematical equations, graphs containing processes with gate decisions or even more complex mathematical objects [24,25,28]. Models should be as accurate as possible in descriptions of the system and validated against enough data in an unbiased and independent manner [26,27,29]. In theory, we can always find a conceptual model behind any diagnostic methodology [18,27]; however, it is important to highlight that there is always a certain degree of uncertainty associated with any prediction generated by any model [29,30]. Thus, the clinical application of a predictive model depends on the evaluated performance during the validation process. For example, a mathematical model can be considered as part of a diagnostic test if the performance exceeds the minimum sensitivity and specificity required by medical authorities [18,29]. Usually, these values are around 97% but can change depending on the country and disease. Otherwise, models fall into a predictive category and should be taken as an indication or tendency for such possibility, where the clinician’s interpretation and judgment is absolutely necessary. In this scenario, the model prediction is useful as supporting information for the clinician, which is always better than relying on chance, intuition or based on the outcomes of previous patients. In general terms, predictive models should be seen as insights that enable a clinician to make a better informed and supported decision [17,18,20].

## 3. Examples of Clinical Applications of Predictive Modelling

There are several types of modelling techniques that can be used in bioinformatics for clinical diagnostics and prognostics. We listed the most frequently used in clinical contexts and describe their basic characteristics in Table 1. The most straightforward application of predictive models is the capacity of generating a prediction for the future [26,29]; this can be applied in the clinical context for the generation of disease prognostics for example for predicting; the emergence of developing a particular genetic disease, disease evolution, impact on life expectancy, impact on the society or even the success of a given treatment [24,25,26,27]. Predicting survival expectancy and response to treatments based on information from a given tumour biopsy characterization is an application that is often attempted by multiple predictive modelling approaches such as statistical, machine learning and logical network modelling [26,31,32,33,34,35,36,37,38]. Another interesting example is the modelling efforts conducted for predicting the impact and control of SARScov2 transmission effects and control during the COVID-19 outbreak [39,40,41,42]. In these works, statistical and Ordinary Differential Equations (ODE) based models have been successfully used for predicting expected peaks of infected, hospitalized and the timings by which the peaks occurred. Further, simulations from Susceptible and Infected ODE models also predicted useful information for the decision of implementing controlling measures that minimize the total of deaths in a certain region [39,40,41].

Predictive models are also useful for the detection of diseases in an early stage, in particular, if current diagnostic methods fail and the treatment efficiency benefits from early detection. One good example of this scenario is the poor detection rates of 40% observed during ovarian cancer screening programs [43,44]; this type of cancer does not show symptoms up to later stages and by then the treatment success is largely compromised. Some statistical and machine learning models have successfully combined multiple biomarkers resulting in surprisingly high sensitivities (>90%) and reasonable specificities (> 80%) which largely outcompete the sensitivities and specificities obtained under current screening programs [23,45]. The application of such modelling approaches at the point-of-care would definitely improve the identification rates at early stages potentially saving thousands of lives of women from ovarian cancer every year.

Another clinical application of predictive models is the generation of insights when the current diagnostic methodologies are too invasive and put at risk the health of the testing subjects [46,47]. One illustrative example of this scenario is the detection of genetic diseases in prenatal screening by amniocentesis and pre-implantation embryo testing using post-freezing PGT-A next-generation sequencing techniques [12,48,49,50,51,52]. In both cases, the procedures for conducting genetic testing are too invasive, compromising the viability of pregnancy and embryo survival during implantation. Machine learning models have been quite successful in predicting aneuploidies from indirect data such as embryo secretome in culture media and urine [48,49]. Impressively, predictive models from mass spectral patterns of secretome have rendered sensitivities very close to the diagnostic level with reasonably tolerable false positive rates, enabling affordable and non-invasive testing [48].

Sequence-based prediction of pathogenic genetic variants (Single Nucleotide Polymorphisms, insertions or deletions) is becoming now a very important modelling application in clinical bioinformatics, in particular for the identification of rare genetic diseases [53,54,55]; these are based on predicting deleterious effects on protein function from gene sequence based on evolutionary conservation of sequence motifs or on machine learning approaches. There are multiple successful examples of models and tools with reasonable sensitivities over 85% such as SIFT, mutation taster, mutation accessor, Fathmn, Phanter and Polyphen-2 [54].

## 4. Choosing the Correct Modelling Framework

Choosing the correct modelling framework is a critical step in developing a suitable model and often is neglected from the beginning [5,56,57]. In most cases, researchers often start from their favourite modelling framework in an attempt to apply it in a given problem; this is not the best policy and resembles the usage of a hammer to perform all construction labour. Ideally, we should first gather the available data, available knowledge of the system we want to model and access which is the best suitable modelling framework for that particular case [27,58]; this is very tedious and theoretical research work but often pays off as it will save time later on by preventing reaching dead ends where models do not describe the systems, cannot be validated or their performances are simply not different from flipping a coin. Here, we briefly describe some advantages and disadvantages of the most promising modelling frameworks with clinical applications.

Machine-learning is a very powerful approach which is ideal for building disease classifiers with yes or no outcomes [8,21,59,60]. For the choice of this approach, it is mandatory to have sufficiently large datasets where the disease outcomes are known [21,60]; this is an absolute requirement for the training and validation of models. Using this approach, it is recommended to try multiple algorithms that have been quite success with biomedical data such as random forests, neural networks, regression models and support vector machines [21,60]. Neural networks (NN) and Recurrent Neural Networks (RNN) are particular important types of machine learning algorithms based on its high efficiency and robustness if well implemented and validated [61,62]; this is particularly important for modelling sequence-based phenotypes with clinical relevance. Hyperparameter exploratory analysis is also a necessary task in this approach, which can be an extensively time-consuming and computationally heavy [59,63]. Most of these algorithms are available in R packages, python libraries and even in Auto ML tools which is a huge advantage that facilitates the implementation of automated workflows for the model generation [63,64]; however, machine learning approaches are “black box” models which are prone to overfitting and artefactual models [21,60,65]. Thus, such modelling frameworks require additional and periodically checking of their reliability; moreover, the absence of knowing the exact rationale behind such prediction with some algorithms may cause difficulties in registration of diagnostic tests and patents.

Statistical models are the most conservative modelling approaches used in clinical contexts [21,66,67]. The development of these types of models relies on the choice of a theoretical statistical model and requires the estimation of its parameters with data. Often this approach is combined with machine learning algorithms for data fitting-based parameter estimation [21]. Statistical models can offer an estimated probability of having or not a particular disease; this brings an advantage over classification models in particular for the scenarios that best describe “gray” zones of uncertainty making them more realistic than the yes/no classification models This type of modelling depends on sample size numbers but often do not require large datasets as in machine learning approaches. Another advantage of this modelling framework is that is simple and has a straightforward implementation in laboratory software tools. A good example of these advantages was capture for the screening of multiple blood disorders using mass spectrometry, where we implemented a cumulative probability function in a laboratory software tool that enables automated estimating of the probability of a patient having a particular type of hemoglobinopathy on a large scale of analysis, applicable to population screenings [22].

Deterministic frameworks such as ODE-based (also called kinetic) and logical modelling are powerful quantitative (kinetic) and discrete modelling approaches (logical) [28,30,68]. Both are by far more descriptive in comparison to statistical and machine learning. The underlying principles of these frameworks rely on the laws of chemistry, physics, biochemical circuits and mathematics, making them more realistic and robust for finding drug targets and predicting therapeutic effects [24,27,28]. For example, exploring kinetic and logical models of signalling pathways in cancer growth and invasion results in predicted effects of drug targets for cell decisions that can be useful for therapy choices and predict cancer aggressiveness and progression [33,34,36,69,70,71]. In contrast with statistical and machine learning, this approach does not rely on sample sizes but requires extensive literature knowledge including knowing parameters and relational laws [27,28]. Developing such models is a huge investment of effort and time-consuming in comparison to the other frameworks as are more complex in terms of variables, and development and require an huge in-depth knowledge of the system; these models may take years to develop and depend on the availability of existing literature data or the capacity to estimate them experimentally [27,28]. In comparison, kinetic models are always preferable to logical as they are more accurate descriptions of the systems and provide a quantitative assessment [28,30,68]. The only advantage of choosing logical modelling is in the case where the kinetic parameters (e.g., rate constants of the processes) are unknown [28].

## 5. Challenges of Clinical Bioinformatics: The Business Perspective

Innovative technology coming from academic research related to clinical bioinformatics is attracting private investment and generate new startups. Most of these startups come from the academic labs which have developed during research projects attractive state-of-the-art bioinformatics algorithms and pipelines that can be applied to a new service or product that potentially can generate growth [3,8]. Upon investment, these startups face a paradigm change that constitutes a huge challenge for both academics that migrate to the industry and investors that need to guarantee the revenues of their investment. One of the main initial issues that most startups are facing is related with software tools and copywrite issues. In bioinformatics, the current way of thinking is based on the usage of command line tools which were developed for academic purposes and are restricted to commercial usage. Sometimes the developed technology utilizes such tools which causes software license issues; this forces startups to either develop their own “in house” workflows almost from scratch which is time-consuming, or buy the respective licenses which in most scenarios can compromise the business sustainability.

Another key issue is the patient’s data; this is often a very sensitive issue which requires following an ethics protocol of personal data protection during data acquisition, storage and analysis [8,72]. Thus, it is absolutely mandatory to implement a secure database system for protecting patients’ personal data and still enabling the bioinformatics pipelines to access some metadata of relevance for conducting the analysis [72]. A simple solution for this could be through using anonymized data pulling during the analysis and automated reporting that can be generated through the usage of secure relational databases.

Code protection is quite trivial in most informatics companies as a standard of best practices but in most academic bioinformatics groups the data is often saved in the postdoc personal computer and publicly available in multiple GitHub accounts. Although this is a severe data security breach it makes it impossible to get copyrights and patents leading to a business loss or a huge shift from original technology [72]. Ideally, the code and also the data should be stored in data centres for ensuring enough security, privacy police and maintenance with proper SSL certificates. Additionally, proprietary code should also be store into private GitHub or GitLab accounts for organizations with suitable ownership of the company and restrictive accessions of developers from both inside and outside of the organization. Both GitHub and GitLab enables such functionalities even for free as this is standard in commercial-based informatics projects and businesses. Ideally, these practices should be taken into account as early as possible, even during the phase of technology development.

Often, the transition from the academical environment to an industry startup environment must follow a huge change in the mind set of researchers; this includes the way to think and work also. From individually tacking a project to teams within compartmentalized projects, to following standard methods of software development like management frameworks like agile and the available implementations such as Kanban board; this last agile implementation is a very popular and flexible solution which is frequently available in many online software tools such as GitHub and Jira. The focus will be no longer, addressing a question and understanding a mechanism. Instead, the focus is finding solutions that are robust and meet company objectives with defined deadlines. One solution to facilitate this is to take advantage of available online courses in the field of informatics that can introduce the best practices to follow for project management and tools; this would help substantially researchers to optimize and adjust their way of working and tackling projects.

Scalability is also another issue to deal, as most of the technology is thought as an analysis service conducted by bioinformaticians as users; this would eventually become saturated because finding bioinformaticians as work force is limited and not an easy task [8,64]. Besides, the cost-effectiveness of the service is also compromised as well the competitiveness simply because bioinformaticians are not cheap labour. In this case, the ideal system would the implementation of automated pipelines that conduct the analysis without human intervention under a software as a service business model (SaaS); this would ensure both scalability and cost-effectiveness as there is only need for a bioinformatics team to maintain and improve the pipelines. Therefore, hiring the correct bioinformatics team is fundamental for the health and growth of the startup; this is often neglected and is indeed a difficult task to find such highly specialized professionals which sometime can be considered as rare unicorns.

## 6. Conclusions and Perspectives

Predictive modelling approaches have a fundamental role in ensuring the applicability of bioinformatics in the clinical context; it is also fundamental to invest in the correct modelling framework for each case and properly integrated with the bioinformatics pipeline and high-throughput technology to ensure the robustness of results given to a clinician and a technological gain in comparison to current methodologies available.

Clinical bioinformatics is still in its initial phase of growth and many startups are only now emerging from new born innovative technology coming from academia. Yet there is a long learning and adaptative process for successfully migrating from an academical mindset towards sustainable clinical bioinformatic services. There are still many challenges to overcome in the future to ensure a successful acceptancy of clinical bioinformatics and its generalization to the point-of-care. The future of clinical bioinformatic may depend on choosing a suitable and modern business model such as SaaS to ensure the sustainability of clinical bioinformatics services. In the future, this would be fundamental for keeping up with a possible scaling up of the demand for bioinformatic services from clinicians. Also keeping up with migration of clinical data to its digital form and becoming compatible with “big data” processing.

## Figures and Tables

**Table 1:** Often used modelling techniques in clinical bioinformatics and their main characteristics.

Modelling Technique	Description	Application	Requirements
Statistical	Scoring and probability functions that assumes a distribution shape or behaviour.	Continuous Quantification	Data for parameter estimation. Depend on sample size.
Kinetic	Solving of systems of nonlinear differential equations. Do not assume any behaviour. Instead relies on rate laws of processes such as chemical reactions.	Binary Classification	Requires reported or estimated kinetic parameter. Do not depend on sample size.
Logical	Solving of logical equations based on predefined rules for each component. Assumes asynchronous or synchronous update schemes.	Binary Classification	Requires relational knowledge of its components. Do not depend on sample size.
Regression	Fitting of an assumed mathematical equation on data. Often are used models that describe a particular assumed data behaviour such as linear, polynomial, exponential, and logistic.	Binary Classification	Data for model fitting. Depend on sample size.
Random Forests	Supervised machine leaning algorithm based on averaging multiple generated decision trees.	Binary Classification	Data for model training and validation. Requires large datasets
Support Vector Machines	Supervised machine leaning algorithm based on clustering algorithms such as principal component analyses.	Binary Classification	Data for model training and validation. Requires large datasets
Neural Networks	Supervised machine leaning algorithm based on defining a set of neuron and layers as model components. Assumes all possible relational interactions between neurons.	Binary Classification	Data for model training and validation. Requires large datasets

## Data Availability

No additional data is available for this commentary.
